# Outcomes of Preterm Infants Admitted to Canadian NICUs Before and During the SARS-COV Pandemic

**DOI:** 10.3390/children12020193

**Published:** 2025-02-06

**Authors:** Marsha Campbell-Yeo, Amy Mireault, Fabiana Bacchini, Marc Beltempo, Prakesh S. Shah, Lynsey Alcock, Jeannette Comeau, Justine Dol, Amy Grant, Jonathan Gubbay, Brianna Hughes, Amos Hundert, Darlene Inglis, Yasmin Lalani, Morgan MacNeil, Thuy Mai Luu, Souvik Mitra, Michael Narvey, Karel O’Brien, Paula Robeson, Michelle Science, Canadian Neonatal Network (CNN) Investigators

**Affiliations:** 1 Dalhousie University, Halifax, NS B3H 4R2, Canada; 2 MOM-LINC Laboratory, IWK Health, Halifax, NS B3K 6R8, Canada; 3 IWK Health, Halifax, NS B3K 6R8, Canada; 4 Canadian Premature Babies Foundation, Toronto, ON M8X 1Y3, Canada; 5 McGill University Health Centre—Montreal Children’s Hospital, Montreal, QC H4A 3J1, Canada; 6 Mount Sinai Hospital, Toronto, ON M5G 1X5, Canada; 7 Department of Pediatrics, Faculty of Medicine, University of Toronto, Toronto, ON M5S 1A1, Canada; 8 Maritime SPOR Support Unit, Halifax, NS B3H 0A2, Canada; 9 BC Children’s Hospital, Vancouver, BC V6H 3N1, Canada; 10 Laboratory Medicine, Department of Pathology, Faculty of Medicine, University of British Columbia, Vancouver, BC V6T 1Z4, Canada; 11 Faculty of Nursing, University of Prince Edward Island, Charlottetown, PE C1A 4P3, Canada; 12 Humber River Health, Toronto, ON M3M 0B2, Canada; 13 Département de Pédiatrie, Centre Hospitalier Universitaire Sainte-Justine, Montréal, QC H3T 1C5, Canada; 14 Department of Pediatrics, Children’s Hospital Research Institute of Manitoba, Winnipeg, MB R3E 3P4, Canada; 15 Children’s Healthcare Canada, Ottawa, ON K2A 3W9, Canada; 16 Sick Kids Hospital, Toronto, ON M5G 1E8, Canada

**Keywords:** SARS-COV, health outcomes, neonatal outcomes, preterm

## Abstract

Background: To better elucidate the impact of the SARS-COV pandemic on neonatal outcomes, we compared the health outcomes of infants born preterm requiring care in a Canadian NICU before and during the SARS-COV pandemic. Methods: Using a retrospective cohort study, infants born between 23 and 32 weeks gestation who were admitted to tertiary Canadian NICUs before and during the pandemic were included. A total of 7280 infants were in the pre-pandemic cohort (admitted 1 April 2018–31 December 2019), and 7088 infants were in the pandemic cohort (admitted 1 April 2020–31 December 2021). The primary outcomes included major morbidity or mortality rates. Care strategies and treatments were compared across the two periods. The relative risk (RR) for the pandemic period, compared to the pre-pandemic period, was calculated using a Poisson regression model, adjusted for identified risk factors. Results: There were no significant differences in infant characteristics between the pre-pandemic and pandemic cohorts. The risk of mortality or major morbidity was comparable before and during the pandemic (37% pre-pandemic, 36% pandemic; RR = 1.01, 95% CI 0.92, 1.01). Individual risks for morbidity and mortality did not differ significantly between periods. There was a clinically significant decline in the receipt of the mothers’ own milk exclusively at discharge during the pandemic (45% before and 37% during; RR 0.85, 95% CI 0.68, 1.06). Conclusions: There were no significant differences in major morbidity or mortality rates in preterm infants between pre-pandemic and pandemic cohorts in Canadian NICUs.

## 1. Introduction

Infants born very preterm (<33 weeks) are at increased risk of short-term morbidities or death [1,2]. In regionalized perinatal care systems, the majority of infants are admitted to a tertiary-level neonatal intensive care unit (NICU), where they are closely monitored by a multidisciplinary team [3]. While there is some evidence to suggest higher preterm birth rates [4], higher NICU admission rates, and a higher likelihood of respiratory distress [5,6,7,8] among birthing parents who test positive for SARS-COV during admission, there are no large-scale studies examining the effects of the SARS-COV pandemic on at-risk neonatal populations. As such, there is an important need to identify the outcomes of these infants to help inform health effects, family needs, and health system planning.

During the pandemic, numerous factors significantly influence neonatal and maternal outcomes. For example, lower rates of health-seeking behaviour and reduced access to care, coupled with changes in care practices, could potentially compromise the quality of neonatal care [9]. Staffing challenges, largely driven by anxiety, stressors, and attrition among healthcare professionals, further contribute to the complexity of providing optimal care [9]. In addition to these challenges, mothers/birth persons and caregivers face elevated stress levels due to uncertainties surrounding concerns about the impact of SARS-COV on their own health and that of their infants and strict parental presence restrictions and policies [10,11,12,13]. In-person parental presence and involvement in infant care was very reduced or absent during SARS-COV, as most NICUs prevented parents from being present in the NICU with their infant or only having one parent present [14]. These stressors, albeit indirect, could potentially have had an effect on the health outcomes of neonates.

While the predominant focus of the evidence is on the negative outcomes, it is essential to acknowledge some potentially positive aspects that may have emerged during the pandemic. Increased awareness of virus transmission led to the implementation of heightened safety and sanitation protocols, including widespread mask usage, of which many of these protocols continue to be implemented in a varying capacity today due to their effectiveness in reducing not just SARS-COV, but the spread of other diseases and viral infections [15].

Given the multitude of factors that deviated from the typical trajectory of maternal and infant care, a thorough evaluation of the outcomes of neonates born during the pandemic is crucial. This assessment aims to uncover any potential impacts, both positive and negative, and inform future healthcare strategies for neonatal care in the context of pandemics caused by respiratory pathogens such as SARS-COV.

### Objective

This study aimed to investigate the health outcomes of preterm infants admitted to Canadian NICUs before and during the SARS-COV pandemic.

## 2. Materials and Methods

### 2.1. Study Design, Participants, and Setting

This was a retrospective cohort study of preterm infants born between 23 and 32 weeks gestational age (GA) and admitted to 1 of the 32 tertiary-level Canadian NICUs participating in the Canadian Neonatal Network (CNN), a national research organization. Infants who were moribund (died at or shortly after birth), who had planned palliative care after birth, or who had missing data (GA, birthweight, admission, or discharge date) were excluded from the study. To ensure a more comparable population, infants with major congenital anomalies were also excluded.

Infants were divided into two cohorts: epoch 1, covering the pre-pandemic period (1 April 2018–31 December 2019), and epoch 2, covering the pandemic period itself (1 April 2020–31 December 2021). We analyzed the differences in neonatal health outcomes between the pre-pandemic and pandemic cohorts.

### 2.2. Outcomes

Primary outcomes included mortality or major morbidity rates, which were chosen based on the incidence and risk for associative long-term neurodevelopmental consequences. Major morbidity was defined as one or more of any of the following criteria: bronchopulmonary dysplasia, severe neurologic injury, necrotizing enterocolitis, severe retinopathy of prematurity, or late-onset sepsis [3]. Bronchopulmonary dysplasia was defined as the need for supplemental oxygen at 36 weeks’ postmenstrual age or at the time of discharge or transfer [16]. We defined severe neurologic injury as grade III or IV intraventricular hemorrhage or cystic periventricular and/or echogenicity [17]. Necrotizing enterocolitis was defined as stage two or greater, diagnosed according to Bell’s criteria [18]. Severe retinopathy of prematurity was defined as stage III or greater in at least one eye according to the international classification [19] or the need for treatment. We defined late-onset sepsis as positive blood or cerebrospinal fluid culture in a symptomatic neonate after 2 days of age.

Secondary outcomes were NICU care practices, interventions, and infant discharge disposition. These outcomes included resuscitation and admissions, interventions and resource use, including the administration of a surfactant, the treatment of patent ductus arteriosus (PDA), respiratory and nutritional support, receipt of mothers’ own milk (MOM), and discharge disposition. Maternal and infant characteristics were collected, including the SNAP-II (Score for Neonatal Acute Physiology II) score. The SNAP-II is a scoring system used to assess the severity of illness in newborns in the NICU and is designed to predict mortality risk based on various physiological parameters. The snap score ranges from 0 to 155, with a score >20 associated with higher odds of mortality and morbidity among very preterm infants [20,21]. 

### 2.3. Data Collection

The CNN database was used to collect demographic characteristics and health outcomes. Across the 32 sites participating in the CNN (https://shorturl.at/wfCCy accessed on 25 January 2025), trained abstractors collected neonatal data from patient charts according to standard definitions and data collection protocols [22]. All patient data were anonymized and inputted electronically into a tailored database with integrated error checks to ensure optimal data quality. The CNN database has been reported to have a high internal consistency and reliability [23]. 

### 2.4. Statistical Analysis

For the primary analyses, we compared neonates born in the pre-pandemic cohort to those admitted throughout the pandemic period cohort. Baseline characteristics were reported for categorical variables using counts and percentages and using the mean standard deviation (SD) for continuous variables. Fisher’s Exact test or chi-square test was used to compare categorical variables. Relative risk (RR) and 95% confidence intervals (CIs) used for categoric outcomes between the pandemic and pre-pandemic cohorts were estimated using robust Poisson regression analysis. A comparison of continuous variables was conducted using Student’s *t*-test (two-sided) if normally distributed and the Mann–Whitney *U* test if not normally distributed. To account for possible clustering at each site, generalized estimated equations were utilized. Relative risks were adjusted for known risk factors for outcomes: gestational age, antenatal steroids exposure, antenatal magnesium sulphate, mode of delivery, multiple births, the Score for Neonatal Acute Physiology version II [SNAP-II] > 20 [21] and small for gestational age (below the 10th percentile for sex and GA based on the populational reference) [24]. The mean difference in discharge weight was estimated using linear regression analysis and was adjusted for patient risk factors (listed above) and site (via GEE). For the other continuous outcomes, median differences were estimated using quantile regression analysis and adjusted for patient risk factors (listed above) and site (fixed effect). Significance was defined as a *p*-value < 0.05. Data management and statistical analyses were performed using SAS, version 9.3 (SAS Institute Inc., Cary, NC, USA).

### 2.5. Sample Size

An initial sample size of at least 5400 infants per group was established to detect a 3% absolute difference in the risk of death or major morbidity with a power of 90% and a type 1 error of 5% using Fisher’s Exact test (assuming a 35% primary outcome rate of mortality/morbidity). Such a sample size would also be able to detect a 1.5% absolute difference in secondary outcomes with lower event rates (such as 5%), a power of 90%, and a type 1 error of 5% using Fisher’s Exact test.

### 2.6. Ethical Approvals

Ethical approval was attained from the IWK Health Research Ethics Board (REB # 1025748) following the authorization of data release from the CNN Executive Committee.

## 3. Results

**Figure 1:**
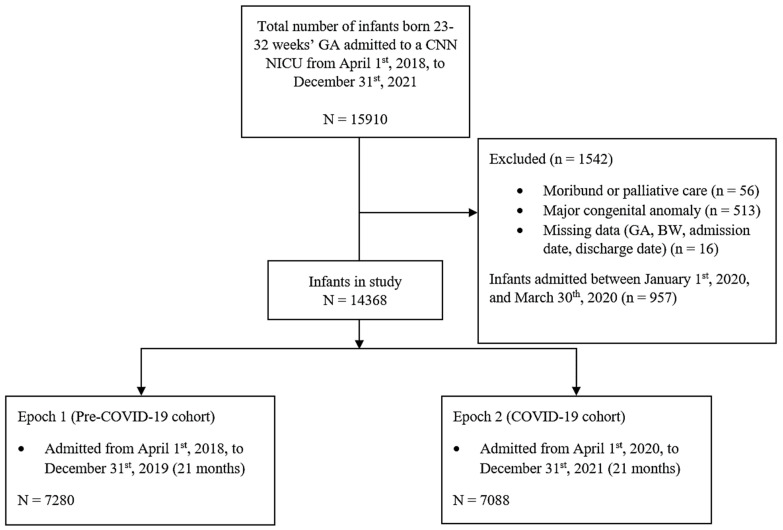
Flow diagram of the study population.

Of the 14,368 neonates included in the study, 7280 infants were admitted during the pre-pandemic cohort (1 April 2018–31 December 2019), and 7088 infants were admitted in the pandemic cohort (1 April 2020–31 December 2021) (Figure 1). Most baseline maternal and infant characteristics were similar across the two cohorts with the exception that the SARS-COV cohort received more antenatal steroids and magnesium sulphate, were slightly older, and had better SNAP scores (Table 1).

Results of the primary and secondary outcomes are reported in Table 2. Infant health outcomes were not significantly different between periods. Given that preterm infants < 29 weeks may have higher risks of adverse outcomes, which statistical adjustment for gestational age may not completely account for, we also conducted sub-group analyses among <29 (Table 3) and 29–32 weeks separately (Table 4).

The incidence of mortality or major morbidity was 37% (2667/7280) in the pre-pandemic cohort and 36% (2561/7088) in the pandemic cohort (adjusted RR = 1.01, 95% CI = 0.92–1.10). Crude and adjusted rates of individual morbidities did not significantly differ between periods. We did not find any significant differences across outcomes in either sub-group.

There was no significant difference in the number of neonates requiring delivery room chest compressions and recorded admission temperature. There was a significant difference in the percentage of infants requiring intubation and assisted ventilation between cohorts of all infants and those born < 29 weeks (crude rates). However, neither of the adjusted rates remained significant. The adjusted median difference (95% CI) in the duration of invasive respiratory support in days was significantly lower in the post-SARS-COV cohort (−0.11 (−0.13, −0.09)) in both the large cohort and <29-week sub-group but not in the 29–32-week sub-group.

The adjusted median difference (95% CI) of the duration of parental nutrition days was significantly less in the SARS-COV cohort (−0.33 (−0.46, −0.2)). There was a non-significant but clinically relevant decrease in the exclusive receipt of mothers’ own milk (MOM) at discharge in the pandemic cohort compared to the pre-SARS-COV cohort (36.3% versus 44%). There was no other significant difference in interventions and resource use between cohorts, nor in discharge disposition.

## 4. Discussion

Our study aimed to compare the neonatal outcomes of very preterm infants born between 23 and 32 weeks admitted to Canadian NICUs before and during the SARS-COV pandemic. This national retrospective cohort study collected data from 14,368 Canadian neonates admitted to the NICU between 1 April 2018 and 31 December 2019, and 1 April 2020 and 31 December 2021, and found no significant differences in neonatal outcomes, care practices and interventions, and discharge dispositions in the NICU between the two different cohorts. Despite the profound effects that the SARS-COV pandemic had on healthcare systems, such as the disruption of healthcare services, including maternity services, changes in standard care protocols and restrictions [25], reduced parental presence [14], and fear-induced reduction in health-seeking behaviours [26,27], our results indicate that the first 20 months of the SARS-COV pandemic had no significant impact on neonatal health outcomes among very preterm infants in Canada. The small improvements noted in the baseline characteristics of SARS-COV may better reflect antepartum care; however, given that the focus of our paper was on neonatal outcomes, we are unable to comment further on this possibility.

Our results augment findings from related studies that evaluated neonatal and maternal outcomes during the SARS-COV pandemic [28,29,30,31,32]. McDonnell et al. conducted a retrospective review in Dublin, focusing on maternal and neonatal outcomes. Gurram et al. carried out a retrospective cohort study involving three Canadian NICUs participating in CNNs, specifically studying neonates born at or beyond 36 weeks. Additionally, Yang et al. conducted a comprehensive living systematic review, encompassing 37 studies and comparing pregnancy and neonatal outcomes before and after the pandemic periods. Importantly, all three studies consistently reported no adverse effects on neonatal outcomes, including stillbirth, prematurity, and neonate gestational weight during the pandemic period [30,31,32]. It is worth noting that these studies primarily examined maternal outcomes and did not specifically focus on preterm infants, distinguishing them from our study.

We compared broader major neonatal morbidities, such as NEC, BPD, and ROP, with very preterm infants before and during the SARS-COV pandemic in a national cohort. Our findings associated with these outcomes are aligned with a cohort study examining neonatal outcomes, specifically in SARS-CoV-2 positive mothers/birthing persons. No association with increased durations of respiratory support, morbidities, mortality, or length of hospital stay was reported among neonates admitted to the tertiary NICU in Sweden or Canada [33]. 

In contrast, a single-centre cohort study conducted by Palizban et al. (2022) in Iran assessed preterm births and major complications of prematurity, including IVH, RDS, ROP, and sepsis, both before and during the SARS-COV pandemic [34]. Their findings demonstrated decreased instances of RDS and an increase in the occurrences of ROP, IVH, and sepsis. The disparities between the two studies, including differences in methodology (one being a single-site cohort study), setting, and contextual factors, such as variations in the reported SARS-COV pandemic waves, procedures, and adherence to hygiene protocols, may have had a direct influence on the reported neonatal outcomes [34].

Furthermore, our results showed no significant differences between care practices and interventions, such as resuscitation and oxygen use, between the two periods. Our findings contrast with those of a retrospective population-based cohort study comparing preterm infants born pre-pandemic between 2015 and 2019 and those born during the pandemic in 2020 in Switzerland using data from the Swiss Federal Statistical Office (FSO) and from SwissNeoNet. In that study, the authors reported higher odds of contracting respiratory distress syndrome (OR 1.6, 95% CI 1.08–2.37) and the need for continuous positive airway pressure (CPAP) (OR 1.39, 95% CI 1.05–1.84) [35]. While it may be possible that RDS could be associated with the pandemic, the higher incidence of CPAP could reflect the trend in practice towards non-invasive ventilation use. Our findings support this possible explanation as we found a decrease in intubation and duration of invasive respiratory days in the post-SARS-COV group.

Similarly, we did not find any difference in the occurrence of NEC in the two cohorts. In two smaller cohort studies examining the potential effect of the pandemic on preterm infants from Italy and Sweden, no effect on the overall incidence of late-onset sepsis nor necrotizing enterocolitis (NEC) was reported [36]. While a significantly lower incidence of NEC at 1.43 vs. 1.94% was reported in the Swedish cohort, there was no difference in the rate of surgical NEC or when adjusted for GA, probiotics, IUGR and chorioamnionitis, or region of birth [35].

We saw a reduction in days of parenteral nutrition, which might simply reflect the recent change in neonatal care practices towards earlier and faster enteral feeding for neonatal care [37]. Despite a short duration of parenteral nutrition, we did not see the expected rise in the use of breastmilk, the recommended source of enteral nutrition in very preterm infants [38]. In contrast, we found a lower rate of receiving exclusive MOM at discharge in the SARS-COV cohort compared to the pre-SARS-COV cohort. The decrease in the receipt of exclusive MOM may be associated with the institution’s restrictive parental presence policies [14], in keeping with significant concerns raised by parents [39,40], as well as decreased MOM support [33]. Findings related to MOM receipt during the pandemic appear mixed with a UK retrospective cohort study of 2073 mother–infant pairs in two NICUs, demonstrating no differences in MOM feeding odds during admission, at discharge, or exclusive MOM feeding at discharge before or during the pandemic [41]. Similarly, a study also reported lower rates of MOM feeding at discharge in Canadian neonates [33]. Of note, in a large global survey of NICU parents’ experiences of parental restrictions during the pandemic, while wide country variations were reported, breastfeeding and the provision of mothers’ own milk, while impacted, did not seem to have been affected to the same degree as parental presence and skin-to-skin contact [11].

We found no significant difference or worsened outcomes during the pandemic. It may be that the SARS-COV pandemic did not influence neonatal outcomes because there was no direct or indirect effect. While staff morale and absenteeism may have been challenging, nursing-to-patient ratios were likely maintained. Unlike in adult care areas, there may have been no or less competition for resources between SARS-COV patients and non-SARS-COV patients.

Our findings also may highlight, like many other countries, the strengths of Canada’s response to the pandemic. Canada implemented stringent public health measures, including the early adoption of social distancing and masking when its case numbers were comparatively lower than other countries, and it attained one of the highest rates of domestic vaccination coverage globally [38,39,40]. In addition, healthcare systems responded by redeploying and acquiring resources (i.e., ventilators) for areas of higher need, such as the NICU and implementing early policies to decrease the spread of infection [38,39]. All these measures may have contributed to Canada’s performance in mitigating the direct effects of the pandemic. These findings also call for commendation for the dedicated healthcare professionals in the NICU who worked diligently under considerable external stress, unwavering in their commitment to providing care for our most fragile preterm infants to neutralize the potential effects of the pandemic.

### Limitations

There are limitations to this study. In the SARS-COV cohort, data regarding the maternal status of SARS-COV diagnosis were not collected. Consequently, we cannot report on the impact of SARS-COV virus positivity on specific outcomes. In a recent Canadian and Swedish analysis, the authors did not show maternal SARS-COV status having an on the outcomes of very preterm infants [33]. However, this study does show a reduction in exposure to mothers’ own milk at discharge. As such, given our study’s limitations, we may not be able to discriminate if the reduction in exposure to mothers’ own milk at discharge was due to the maternal SARS-COV status or peri-pandemic practice changes (e.g., reduction in breastfeeding support and parent restriction policies). Further research is needed to elucidate this specific effect across multiple possible variables. Of note, in a national consensus statement, parent and healthcare provider priorities were highlighted, including the parent’s presence in the NICU in the post-pandemic recovery period, ensuring ways to support maternal presence, access to breast pumps, and breastfeeding supports; these constituted the six strong recommendations identified [41]. 

While the strengths of our study include the large sample size, quality of the national database, and a wide array of important outcomes across the incidence of mortality, major morbidities, resuscitation, interventions, resources and discharge, our findings do not reflect the possible alteration of other outcomes. For example, we were unable to report on the possible longer-lasting effects of parent–infant separation or reduced infant stimulation that may have been affected by the institution’s severe restriction policies on the presence of parents during the pandemic. In a recent study published in *Children* examining the neurobiological importance of the role of tactile experiences and skin-to-skin contact in preterm infants, it was reported that affective touch by parents, mainly through the activation of tactile C-fibres, improves caregiver–infant bonding, reduces stress responses, and supports neurodevelopment in preterm infants [42]. 

Moreover, given the timing of data collection, the findings do not consider possible differences in outcomes related to the Omicron Variant of the SARS-CoV-2 or the influence of broad vaccination uptake. Instead, this study concentrated on the broader potential impacts of the pandemic on infants born very preterm in Canadian NICUs rather than specifically examining the effects of the SARS-COV virus itself. Also, it is essential to note that this study represents national data solely from Canada and that this study only focused on NICU care outcomes and did not assess the overall populational impacts of SARS-COV on the rates of preterm births. The observed outcomes may not apply to other countries due to variations in resources, regulations, and protocols unique to each nation.

## 5. Conclusions

This retrospective cohort study, comparing preterm infants born between 23 and 32 weeks GA before and during the SARS-COV pandemic in Canada, did not find any significant associations between the SARS-COV cohorts and neonatal morbidities, mortality, resuscitation, interventions, resource use, or length of hospital stay. The lower receipt of exclusive MOM at discharge in the SARS-COV sub-group of neonates, although not statistically significant, warrants further study. The findings suggest that the SARS-COV pandemic had no significant impact on the immediate neonatal health outcomes of very preterm infants in Canadian NICUs. Further research examining the possible longer-lasting effects on infant neurodevelopment and parent well-being is also warranted.

## Figures and Tables

**Table 1:** Baseline maternal and infant characteristics.

Variables	Pre-SARS-COV Cohort (*n* = 7280)	SARS-COV Cohort (*n* = 7088)	*p*-Value
**Maternal Characteristics**			
Age, years ^1^	31.7 (5.8)	31.9 (5.6)	0.02
Parity, >1	1615 (24.5)	1639 (25.1)	0.40
Smoking during pregnancy	8 (0.1)	5 (0.1)	0.43
Diabetes	1220 (17.5)	1247 (18.4)	0.15
Hypertension	1484 (21.0)	1549 (22.5)	0.03
Antenatal steroids	6431 (89.4)	6341 (90.6)	0.02
Antenatal MgSO_4_	4610 (66.8)	5194 (76.8)	<0.01
Rupture of membranes ≥24 h	1731 (25.1)	1676 (25.4)	0.71
Vertex presentation	4396 (64.1)	4323 (64.1)	0.99
Cesarean section	4655 (64.0)	4648 (65.7)	0.04
Multiple births	2168 (29.8)	1982 (28.0)	0.02
**Infant Characteristics**			
Gestational age, weeks ^1^	28.9 (2.6)	29.0 (2.6)	0.09
Gestational groups			
<26	997 (13.7)	930 (13.12)	0.17
26–28 weeks	1830 (25.14)	1723 (24.31)	
29–30 weeks	1823 (25.04)	1808 (25.51)	
30–31 weeks	1424 (19.56)	1475 (20.81)	
Birth weight, grams ^1^	1326 (460)	1335 (458)	0.23
Head circumference, cm ^1^	27.0 (3.1)	27.0 (3.1)	0.36
Male, sex	4040 (55.5)	3908 (55.2)	0.68
Small for gestational age	729 (10.0)	746 (10.5)	0.31
SNAP-II score ^2^	5 (0, 14)	5 (0, 14)	0.10
SNAP-II score > 20	1007 (14.0)	885 (12.6)	0.02
Apgar at 5 min ^2^	8 (6, 9)	8 (6, 9)	0.13
Apgar at 5 min < 7	2015 (28.1)	1913 (27.4)	0.35
Outborn	1009 (13.9)	996 (14.1)	0.74

Data are expressed as n (%) unless otherwise specified. ^1^ Mean (standard deviation). ^2^ Median (interquartile range).

**Table 2:** Primary and secondary neonatal outcomes for infants born <33 weeks.

Variables	Pre-SARS-COV Cohort (*n* = 7280)	SARS-COV Cohort (*n* = 7088)	Relative Risk/Median or Mean Difference (95% CI)	Adjusted Relative Risk/Median or Mean Difference (95%CI)
**Neonatal outcomes** **Relative Risk and 95% CI**				
Mortality or major morbidity %	2667/7280 (36.6)	2561/7088 (36.1)	0.99 (0.96, 1.05)	1.01 (0.92, 1.10)
Mortality %	433/7280 (6.0)	420/7088 (5.9)	1.00 (0.93, 1.07)	1.11 (0.97, 1.27)
BPD ^1^ %	1895/6846 (27.7)	1812/6670 (27.2)	0.99 (0.95, 1.02)	1.01 (0.90, 1.14)
Severe brain injury %	447/6039 (7.4)	453/5929 (7.6)	1.02 (0.95, 1.09)	1.10 (0.98, 1.23)
NEC ^2^, stage ≥ 2 %	255/7279 (3.5)	248/7082 (3.5)	1.00 (0.92, 1.09	1.01 (0.87, 1.18)
ROP ^3^, stage ≥ 3 or treated %	305/7264 (4.2)	276/7063 (3.9)	0.96 (0.89, 1.04)	0.96 (0.82, 1.12)
Late-onset sepsis %	677/7280 (9.3)	624/7088 (8.8)	0.97 (0.92, 1.03)	0.95 (0.85, 1.06)
**Resuscitation/admission in the birth unit**				
Intubation (%)	1837/7280 (25.2)	1566/7085 (22.1)	0.92 (0.89, 0.95)	0.90 (0.78, 1.04)
Chest compressions/epinephrine (%)	222/7280 (3.1)	190/7088 (2.7)	0.94 (0.86, 1.03)	0.98 (0.82, 1.18)
Normal admission temperature ^4^ (%)	2543/7156 (35.5)	2521/6962 (36.2)	1.01 (0.98, 1.05)	1.03 (0.95, 1.10)
**Interventions and resource use**				
Receipt of surfactant at any time (%)	3401/7280 (46.7)	3236/7088 (45.7)	0.98 (0.95, 1.01)	0.99 (0.94, 1.04)
Treated PDA ^5^ (%)	842/2003 (42.0)	757/1927 (39.3)	0.95 (0.89, 1.01)	0.93 (0.84, 1.04)
Air leak requiring treatment ^6^ (%)	274/7280 (3.8)	245/7088 (3.5)	0.96 (0.88, 1.04)	0.96 (0.82, 1.13)
Corrected gestational age at discharge among survivors (weeks) (%)	35 (33, 38)	36 (33, 38)	0.14 (−0.03, 0.32)	0.14 (0.05, 0.24)
Any MOM ^7^ receipt at discharge (%)	5118/7280 (70.3)	5066/7088 (71.5)	1.03 (0.99, 1.06)	1.01 (0.97, 1.04)
Exclusive MOM ^7^ receipt at discharge (%)	3244/7280 (44.6)	2643/7088 (37.3)	0.86 (0.84, 0.89)	0.85 (0.68, 1.06)
			**Median difference and 95% CI**	
Duration of invasive respiratory support, days (IQR)	0 (0, 3)	0 (0, 2)	0 (0, 0)	−0.11 (−0.13, −0.09)
Duration of non-invasive respiratory support, (IQR)	5 (2, 20)	7 (2, 22)	2 (1.16, 2.84)	1.00 (0.76, 1.24)
Duration of any respiratory support, days (IQR)	12 (4, 47)	14 (4, 46)	2 (0.56, 3.44)	1.38 (0.9, 1.87)
Duration of parenteral nutrition, days (IQR)	7 (5, 13)	7 (4, 13)	0 (−0.28, 0.28)	−0.28 (−0.41, −0.16)
Length of stay in all patients, days (IQR)	36 (16, 66)	37 (16, 67)	1 (−0.69, 2.69)	1 (0.34, 1.66)
Length of stay in survivors, days (IQR)	38 (18, 68)	39 (19, 69)	1 (−0.77, 2.77)	1 (0.44, 1.55)
			**Mean difference and 95% CI**	
Duration of oxygen supplementation, days (mean)	2 (0, 23)	2 (0, 21)	0 (0, 0)	0 (−0.27, 0.27)
Discharge weight among those discharged home, grams (mean)	2913 (822)	2902 (788)	−11.15 (−53.42, 31.12)	−5.95 (−77.98, 66.07)
**Discharge disposition** ***p*-value**				
Death	433/7280 (6.0)	420/7088 (5.9)		0.21
Home	2836/7280 (39.0)	2769/7088 (39.1)		
Community Hospital	3990/7280 (54.8)	3890/7088 (54.9)		
Other	21/7280 (0.3)	9/7088 (0.1)		

Models adjusted for gestational age, antenatal steroids exposure, antenatal MgSO_4_, mode of delivery, multiple births, SNAP-II score > 20 and small for gestational age and Generalized Estimation Equations (GEE) approach to account for the clustering within each site. Mean difference in discharge weight was estimated using linear regression analysis and adjusted for patient risk factors (listed above) and site (via GEE). For the other continuous outcomes, median differences were estimated using quantile regression analysis and adjusted for patient risk factors (listed above) and site (fixed effect). ^1^ Bronchopulmonary dysplasia; ^2^ Necrotizing enterocolitis; ^3^ Retinopathy of prematurity; ^4^ Between 36.5 and 37.5 degrees Celsius; ^5^ Patent ductus arteriosus; ^6^ Needle drainage or chest tube; ^7^ Mothers’ own milk.

**Table 3:** Primary and secondary neonatal outcomes in the sub-group of infants born <29 weeks.

Variables	Pre-SARS-COV Cohort (*n* = 2827)	SARS-COV Cohort (*n* = 2653)	Relative Risk/Median or Mean Difference (95% CI)	Adjusted Relative Risk/Median or Mean Difference (95%CI)
**Neonatal outcomes** **Relative Risk and 95% CI**				
Mortality or major morbidity %	1931/2827 (68.3%)	1784/2653 (67.2%)	0.98 (0.92, 1.03)	0.98 (0.94, 1.03)
Mortality %	378/2827 (13.4%)	364/2653 (13.7%)	1.01 (0.94, 1.09)	1.08 (0.93, 1.25)
BPD ^1^ %	1365/2463 (55.4%)	1250/2298 (54%)	0.98 (0.93, 1.04)	0.99 (0.91, 1.07)
Severe brain injury %	356/2470 (13%)	334/2575 (13%)	1 (0.92, 1.09)	1.04 (0.91, 1.17)
NEC ^2^, stage ≥ 2 %	209/2826 (7.4%)	187/2649 (7.06%)	0.98 (0.89, 1.08)	0.94 (0.82, 1.09)
ROP ^3^, stage ≥ 3 or treated %	294/2823 (10.4%)	265/2641 (10%)	0.98 (0.9, 1.07)	0.96 (0.83, 1.13)
Late-onset sepsis %	570/2827 (20.2%)	494/2653 (18.6%)	0.95 (0.9, 1.02)	0.9 (0.8, 1)
**Resuscitation/admission in the birth unit**				
Intubation (%)	1375/2827 (48.6%)	1133/2651 (42.7%)	0.89 (0.85, 0.94)	0.89 (0.76, 1.03)
Chest compressions/epinephrine (%)	136/2827 (4.8%)	129/2653 (4.9%)	1 (0.89, 1.13)	1.09 (0.89, 1.33)
Normal admission temperature ^4^ (%)	1049/2768 (37.9%)	1051/2599 (40.4%)	1.05 (1, 1.11)	1.08 (1, 1.16)
**Interventions and resource use**				
Receipt of surfactant at any time (%)	2113/2827 (74.7%)	1963/2653 (74%)	0.98 (0.92, 1.04)	0.99 (0.95, 1.03)
Treated PDA ^5^ (%)	776/1573 (49.3%)	685/1461 (46.9%)	0.95 (0.89, 1.02)	0.94 (0.84, 1.05)
Air leak requiring treatment ^6^ (%)	147/2827 (5.2%)	145/2653 (5.5%)	1.03 (0.91, 1.15)	1.1 (0.89, 1.37)
Any MOM ^7^ receipt at discharge (%)	1680/2827 (59.43%)	1619/2653 (61.03%)	1.03 (0.98, 1.09)	1.02 (0.97, 1.08)
Exclusive MOM ^7^ receipt at discharge (%)	1284/2827 (45.42%)	1032/2653 (38.9%)	0.88 (0.84, 0.93)	0.86 (0.7, 1.06)
			**Median difference and 95% CI**	
Duration of invasive respiratory support, days (IQR)	3 (0, 16)	1 (0, 9)	−2 (−3, −0.99)	−1.5 (−1.74, −1.27)
Duration of non-invasive respiratory support, (IQR)	22 (8, 36)	24 (9, 36)	2 (0.52, 3.48)	0.83 (−0.03, 1.69)
Duration of any respiratory support, days (IQR)	53 (31, 86)	53 (32, 84)	0 (−2.45, 2.45)	1.5 (−0.23, 3.23)
Duration of parenteral nutrition, days (IQR)	13 (8, 26)	13 (8, 25)	0 (−1.17, 1.17)	−0.81 (−1.26, −0.37)
Length of stay in all patients, days (IQR)	72 (42, 105)	72 (43, 103)	0 (−3.26, 3.26)	0.17 (−1.41, 1.75)
Length of stay in survivors, days (IQR)	79 (53, 109)	80 (54, 108)	1 (−1.96, 3.96)	1.5 (−0.01, 3)
			**Mean difference and 95% CI**	
Duration of oxygen supplementation, days (mean)	32 (7, 70)	31 (6, 67)	−1 (−4.6, 2.6)	−1.93 (−3.38, −0.48)
Discharge weight among those discharged home, grams (mean)	3384.9 (927.8)	3326 (887.8)	−58.85 (−135.95, 18.25)	−62.83 (−153.38, 27.74)
**Discharge disposition** ***p*-value**				
Death	378/2827 (13.37%)	364/2653 (13.72%)		0.53
Home	1094/2827 (38.7%)	1058/2653 (39.88%)		
Community Hospital	1343/2827 (47.51%)	1224/2653 (46.14%)		
Other	12/2827 (0.42%)	7/2653 (0.26%)		

Models adjusted for gestational age, antenatal steroids exposure, antenatal MgSO_4_, mode of delivery, multiple births, SNAP-II score > 20 and small for gestational age and Generalized Estimation Equations (GEE) approach to account for the clustering within each site. Mean difference in discharge weight was estimated using linear regression analysis and adjusted for patient risk factors (listed above) and site (via GEE). For the other continuous outcomes, median differences were estimated using quantile regression analysis and adjusted for patient risk factors (listed above) and site (fixed effect). ^1^ Bronchopulmonary dysplasia; ^2^ Necrotizing enterocolitis; ^3^ Retinopathy of prematurity; ^4^ Between 36.5 and 37.5 degrees Celsius; ^5^ Patent ductus arteriosus; ^6^ Needle drainage or chest tube; ^7^ Mothers’ own milk.

**Table 4:** Primary and secondary neonatal outcomes in the sub-group of infants born 29–32 weeks.

**Variables**	**Pre-SARS-COV Cohort** **(*n* = 4453)**	**SARS-COV** **Cohort** **(*n* = 4435)**	**Relative Risk/Median or Mean Difference** **(95% CI)**	**Adjusted Relative Risk/Median or Mean Difference** **(95%CI)**
**Neonatal outcomes** **Relative Risk and 95% CI**				
Mortality or major morbidity %	736/4453 (16.53)	777/4435 (17.52)	1.04 (0.98, 1.1)	1.09 (0.88, 1.33)
Mortality %	55/4453 (1.24)	56/4435 (1.26)	1.01 (0.84, 1.22)	1.27 (0.9, 1.77)
BPD ^1^ %	530/4383 (12.09)	12.85 (562/4372)	1.04 (0.97, 1.11)	1.08 (0.84, 1.39)
Severe brain injury %	91/3300 (2.76)	119/3354 (3.55)	1.15 (0.98, 1.34)	1.32 (1.07, 1.61)
NEC ^2^, stage ≥ 2 %	46/4453 (1.03)	61/4433 (1.38)	1.17 (0.94,1.45)	1.42 (0.96, 2.1)
ROP ^3^, stage ≥ 3 or treated %	11/4441 (0.25)	11/4422 (0.25)	1 (0.66, 1.52)	0.91 (0.41, 2.02)
Late-onset sepsis %	107/4453 (2.4)	130/4435 (2.93)	1.11 (0.97, 1.28)	1.28 (0.96, 1.73)
**Resuscitation/admission in the birth unit**				
Intubation (%)	462/4453 (10.38)	433/4434 (9.77)	0.97 (0.9, 1.03)	0.94 (0.77, 1.15)
Chest compressions/epinephrine (%)	86/4453 (1.93)	61/4435 (1.38)	0.85 (0.74, 0.98)	0.8 (0.58, 1.09)
Normal admission temperature ^4^ (%)	1494/4388 (34.05)	1470/4363 (33.69)	0.99 (0.95, 1.04)	0.98 (0.9, 1.06)
**Interventions and resource use**				
Receipt of surfactant at any time (%)	1288/4453 (28.92)	1273/4435 (28.7)	0.99 (0.95, 1.04)	1 (0.92, 1.09)
Treated PDA ^5^ (%)	66/430 (15.35)	72/466 (15.45)	1 (0.83, 1.21)	0.98 (0.66, 1.45)
Air leak requiring treatment ^6^ (%)	127/4453 (2.85)	100/4335 (2.25)	0.89 (0.79, 1)	0.79 (0.59, 1.05)
Any MOM ^7^ receipt at discharge (%)	3438/4453 (77.21)	3447/4435 (77.72)	1.01 (0.97, 1.07)	1 (0.97, 1.03)
Exclusive MOM ^7^ receipt at discharge (%)	1960/4453 (44.02)	1611/4435 (36.32)	0.85 (0.82, 0.89)	0.84 (0.65, 1.09)
			**Median difference and 95% CI**	
Duration of invasive respiratory support, days (IQR)	0 (0, 1)	0 (0, 0)	0 (0, 0)	0(0,0)
Duration of non-invasive respiratory support, (IQR)	3 (1, 7)	4 (2, 9)	1 (1, 1)	0.75(0.59, 0.91)
Duration of any respiratory support, days (IQR)	5 (2, 13)	7 (2, 9)	2 (1.23, 2.77)	1 (0.82, 1.18)
Duration of parenteral nutrition, days (IQR)				
Length of stay in all patients, days (IQR)	6 (4, 8)	6 (3, 8)	0 (0, 0)	−0.36 (−0.5, −0.23)
Length of stay in survivors, days (IQR)	25 (11, 41)	26 (12, 43)	1 (−0.31, 2.31)	0.6 (0.02, 1.18)
Duration of invasive respiratory support, days (IQR)	25 (12, 42)	26 (12, 43)	1 (−0.61, 2.61)	0.6 (0.01, 1.19)
			**Mean difference and 95% CI**	
Duration of oxygen supplementation, days ^2^	1 (0, 3)	1 (0, 3)	0 (0, 0)	0 (−0.03, 0.03)
Discharge weight among those discharged home, grams (mean)	3384.85 (927.8)	3326 (887.8)	25.14 (−13.68, 63.94)	34.3 (−38.72, 107.32)
**Discharge disposition *p*-value**				
Death	55/4453 (1.24)	56/4435 (1.26)	25.14 (−13.68, 63.96)	0.18
Home	1742/4453 (39.12)	1711/4435 (38.58)		
Community Hospital	2647/4453 (59.44)	2666/4435 (60.11)		
Other	9/4453 (0.2)	2/4435 (0.05)		

Models adjusted for gestational age, antenatal steroids exposure, antenatal MgSO_4_, mode of delivery, multiple births, SNAP-II score > 20 and small for gestational age and Generalized Estimation Equations (GEE) approach to account for the clustering within each site. Mean difference in discharge weight was estimated using linear regression analysis and adjusted for patient risk factors (listed above) and site (via GEE). For the other continuous outcomes, median differences were estimated using quantile regression analysis and adjusted for patient risk factors (listed above) and site (fixed effect). ^1^ Bronchopulmonary dysplasia; ^2^ Necrotizing enterocolitis; ^3^ Retinopathy of prematurity; ^4^ Between 36.5 and 37.5 degrees Celsius; ^5^ Patent ductus arteriosus; ^6^ Needle drainage or chest tube; ^7^ Mothers’ own milk.

## Data Availability

The datasets generated during this study are derived from the Canadian Neonatal Network (CNN). CNN data are available upon reasonable request and adherence to data release processes.

## Abbreviations

Acronym	Definition
BPD	Bronchopulmonary dysplasia
CNN	Canadian Neonatal Network
CI	Confidence interval
CPAP	Continuous positive airway pressure
GA	Gestational age
IUGR	Intrauterine Growth Restriction
IVH	Intraventricular hemorrhage
MOM	Mother’s own milk
NEC	Necrotizing enterocolitis
NICU	Neonatal intensive care unit
PDA	Patent ductus arteriosus
RDS	Respiratory distress syndrome
ROP	Retinopathy of prematurity
RR	Relative risk
SNAP-II	Score for Neonatal Acute Physiology II
SD	Standard deviation
FSO	Swiss Federal Statistical Office
